# Critical Roles of E2F3 in Growth and Musculo-skeletal Phenotype in Mice

**DOI:** 10.7150/ijms.39068

**Published:** 2019-10-21

**Authors:** Hae-Rim Kim, Faiz Ur Rahman, Kwang-Soo Kim, Eun-Kyeung Kim, Sang-Mi Cho, Kihoon Lee, Ok-sung Moon, Young-won Seo, Won-Kee Yoon, Young-Suk Won, Hoyoung Kang, Hyoung-Chin Kim, Ki-Hoan Nam

**Affiliations:** 1 Laboratory Animal Resource Center, Korea Research Institute of Bioscience and Biotechnology, Yeonjudanji-ro 30, Chungbuk 28116, Korea; 2 Department of Animal Science and Technology, Chung-Ang University, Seodong-daero 4726, Gyeonggi 17546, Korea

**Keywords:** *E2f3^+/^*^-^ Knockout, Development, Growth retardation, skeletal imperfection, Mouse phenotype

## Abstract

E2F3, a member of the E2F family, plays a critical role in cell cycle and proliferation by targeting downstream, retinoblastoma (RB) a tumor suppressor family protein. The purpose of this study, was to investigate the role and function of E2F3 *in vivo*. We examined phenotypic abnormalities, by deletion of the *E2f3* gene in mice. Complete ablation of the E2F3 was fully penetrant, in the pure C57BL/6N background. The *E2f3^+/^*^-^ mouse embryo developed normally without fatal disorder. However, they exhibited reduced body weight, growth retardation, skeletal imperfection, and poor grip strength ability. Findings suggest that E2F3 has a pivotal role in muscle and bone development, and affect normal mouse growth.

## Introduction

E2F transcription factors (E2Fs) are found in most cell types of the body and contribute to cell cycle progression, cell proliferation, differentiation, and apoptosis processes 1. These E2F transcription factors are potent regulators of a variety of genes, including cell-cycle checkpoints controlling genes in mammalian cells 2. Until now, eight members have been characterized, E2F1 to 8, and they are generally classified into transcriptional activators (E2F1-3) and repressors (E2F4-8) 3, 4. Some of these E2F family members have also a key role, in myeloid development and cardiac neovascularization 5, 6.

For transcriptional regulation, dimerization partner (DP) family and retinoblastoma (RB) family collaborate with E2Fs, by binding to E2F proteins. DP proteins identified DP1-3 form a heterodimer with an E2F, and allow it to bind to target promoters 7, 8. RB family (pRB, p107, and p130) can bind to activation domains on the E2F1-5, and formed E2F/RB complex 1. This complex on the promoters gives rise to repression of target genes in a cell cycle dependent manner, through epigenetic modification as well as by physical blocking 9-11. Not all functions of the E2Fs are Rb-dependent. E2F3 protein also has functions, unrelated to Rb proteins 12, 13.

The E2F3 is expressed ubiquitously, like other E2F family members 14. However, unlike other E2F family members, the *E2F3* locus encodes two gene products, E2F3a and E2F3b, originating from two different promoters. They share the most coding sequence, such as the DNA binding domain and activation domain, as they possess a unique first exon 15. Two types of the E2F3 isoform are different in expression, because of distinct promoters. E2F3a protein, the long one, is produced like E2F1 and E2F2. E2F3a promoter is expressed highly at G1/S transition, and is regulated by E2F-mediated negative feedback and Myc protein. In contrast, E2F3b promoter is not affected by E2F/Myc-mediated regulation mechanisms, and remains active throughout the cell cycle 15, 16.

E2F3 protein is a key factor in overall biological functions, as it regulates cell cycle progression. Additionally, E2F3 has played a key role in diverse biological processes such as lens development, cardiac neovascularization, DNA damage responses, neuronal migration, and myogenesis 6, 13, 17-21. Dysregulation of E2F3 is closely related to carcinogenesis, and recent studies confirmed that overexpression of miRNAs targeting E2F3, inhibits cell migration and proliferation in many tumors 22-28. E2F3 is considered a promising prognostic marker in specific carcinomas 27, 29, 30.

Given the significance of the E2F3 in key biological processes, we showed its effects on mouse phenotype using *in vivo* study. In this study, we investigated phenotypes of *E2f3* null mutant (*E2f3*^-/-^) mouse in the C57BL/6N background. All *E2f3*^-/-^ embryos were dead in the uterus or soon after birth, and we only obtained hetero mutant (*E2f3*^+/-^) mice. Also, we observed growth retardation and musculo-skeletal imperfection in *E2f3*^+/-^ mice, supporting the key role of E2F3 after birth.

## Materials and Methods

### Generation of E2f3^+/-^ mice

E2f3 gene knockout mouse was produced by Korea Mouse Phenotyping Center (KMPC) using a mouse embryonic stem cell clone with mutant E2f3 gene (*E2f3^tm1a(KOMP)Wisi^*) (EPD0034_2_C11, KOMP repository, CA, USA) obtained from the Mouse Biology Program at UC Davis. We produced the tm1b mutant mouse from the tm1a mutant mouse using HTN-Cre protein (HTN-Cre, Excellgen, USA) as described in the previous paper 31. The tm1b genotype mice, were expanded for all studies. The care and use of all mice used in this study, were in accordance with the IACUC at KRIBB (KRIBB-AEC-17050).

### Body weight curve of wild type and E2f3^+/-^ mice

All mice obtained by mating female and male *E2f3*^+/-^ mice were genotyped at 3 weeks of age. After grouping according to their sex and genotype, weekly body weight measurements were conducted for all the animals from 4 to 16 weeks of aged.

### Dual energy X-ray absorptiometry (DEXA) and X-ray

DEXA and X-ray analysis were conducted at age 14 weeks in mice. The mice were anesthetized with intraperitoneal injection of 1.2% avertin solution, before these analyses. In the Dexa, Lunar Piximus II (GE Medical Systems, Wisconsin, USA) was used and all body composition parameters were obtained with whole body excluding the skull area. For X-ray analysis, Faxitron X-ray system (Model MX-20, Wheeling, IL, USA) was used and exposure X-ray dose per mouse was 300µSv. Six X-ray images per mouse were analyzed: Dorso-ventral (whole body), Lateral (whole body), Dorso-ventral (upper body), Lateral (head), Dorso-ventral (head), and Dorsoventral (lower body).

### Grip strength test

Forelimb strength and combined forelimb/hind limb strength were measured, using the GRS meter apparatus (Chatillon, HMGU plate, USA) with 9-week-old animals. Three trials were conducted in succession, to measure forelimb strength. After a five-minute interval, three successive trials for measuring combined forelimb and hind limb grip strength were conducted.

### Hematological assessment

At age 16 weeks, all the mice were autopsied after anesthesia with intraperitoneal injection of avertin. Before abdominal opening, blood was collected through a heart puncture with EDTA-treated tube (BD Microtainer, NJ, USA). Blood was used for hematology, blood chemistry. Hematological analysis was performed with a Cell-Dyn 3700 hematology analyzer (Abbott Laboratories, IL, USA). Blood biochemistry was conducted with a Hitachi 7020 automatic analyzer.

### Fluorescence assisted cell sorting (FACS) analysis

Splenocytes were obtained from the spleen obtained from autopsy at age 16 weeks, and were stained with fluorescence labeled cell surface antibodies to analyze immune cell subsets. Cells which were surface stained, were analyzes with FACSAria III (BD Bioscience, San Jose, CA, USA). Cell surface staining was performed with antibodies as follow: Anti-mouse CD4 PE-Cyanine5 (#15-0042), Anti-mouse CD5 APC-eFluor® 780 (#47-0051), Anti-mouse CD8a FITC (#11-0081), Anti-mouse Ly-6G (Gr-1) FITC (#11-5931), Anti-mouse Ly-6C APC (#17-5932), Anti-mouse CD11b APC (#17-0112), Anti-mouse CD11c FITC (#11-0114), Anti-mouse CD11c PE-Cyanine5 (#15-0114), Anti-mouse CD11c Alexa Fluor® 700 (#56-0114), Anti-mouse CD19 PE-Cyanine5 (#15-0193), Anti-mouse CD19 PE-eFluor® 610 (#61-0193), Anti-mouse CD19 Alexa Fluor® 700 (#56-0193), Anti-mouse NK1.1 PE-eFluor® 610 (#61-5941) and Anti-mouse MHC Class II (I-A/I-E) PE-Cyanine7 (#25-5321), all from eBioscience (San Diego, CA, USA).

### Statistical analysis

Data were presented as the mean and standard deviation. All statistical differences between groups were analyzed by Student's t-test using a statistical program (GraphPad Prism 7.04, San Diego, CA). P<0.05 was considered as significant.

## Results

### E2F3 is critical for embryo survival and E2f3^-/-^ neonates died in early development stage

To validate the role of E2F3 in mouse development, we mated the *E2f3* heterozygous mutant C57BL/6N mice. However, we couldn't observe viable *E2f3*^-/-^ neonates, only *E2f3*^+/+^ and *E2f3*^+/-^animals were obtained (Table 1). In the previous study that investigated viability of the E2f3 mutant mouse, all the *E2F3*^-/-^ 129/Sv embryos were dead. But C57BL/6
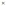
129/Sv mixed mice survived partially, suggesting that *E2f3* is an essential gene for embryonic viability in a strain-specific manner 19. Our data also indicate that deletion of *E2f3* affect embryo survival in C57BL/6N background and that the heterozygosity of *E2F3* gene is enough for the survival in the mice.

### *E2f3^+/-^* mice have lower body weight than wild type mice, suggesting its role in development

**Figure 1:**
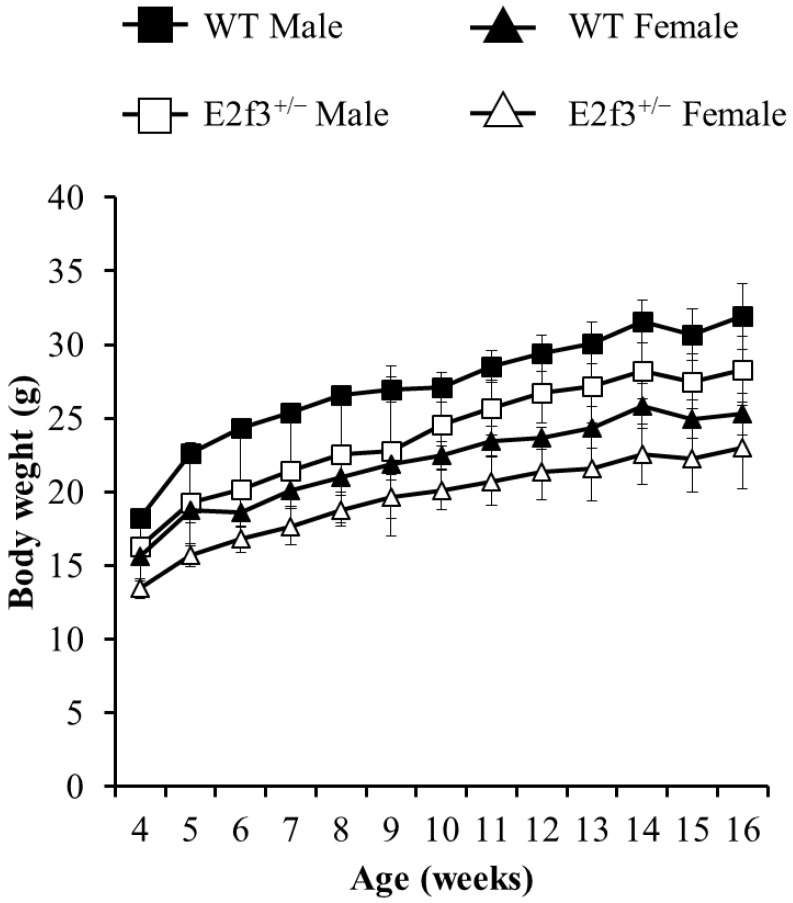
Weight curve of wild-type and *E2f3*^+/-^ male/female mice during 16 weeks. Body weight was measured once a week from age four-16 weeks. During the whole observation periods, body weight of* E2f3*^+/-^ Male (open squares, n = 8), was significantly lower than that of WT Male (filled squares, n = 8). Likewise, body weight of *E2f3*^+/-^ Female (open triangles, n = 8), was significantly lower than that of WT Female (filled triangles, n = 8).

Although *E2f3*^+/-^ animals were born ordinarily, they showed immature phenotype. We conducted various phenotypic analysis for *E2f3*^+/-^ animals, to investigate effects of partial *E2f3* defects after birth. Weekly body weight measurements were conducted, after weaning until age 16 weeks. Our data indicates that female and male *E2f3*^+/-^ mutant mice had shown significantly lower body weight than wild mice throughout the measurement period, suggesting E2f3 is responsible for development as well as maturation in mice (Fig. 1).

**Figure 2:**
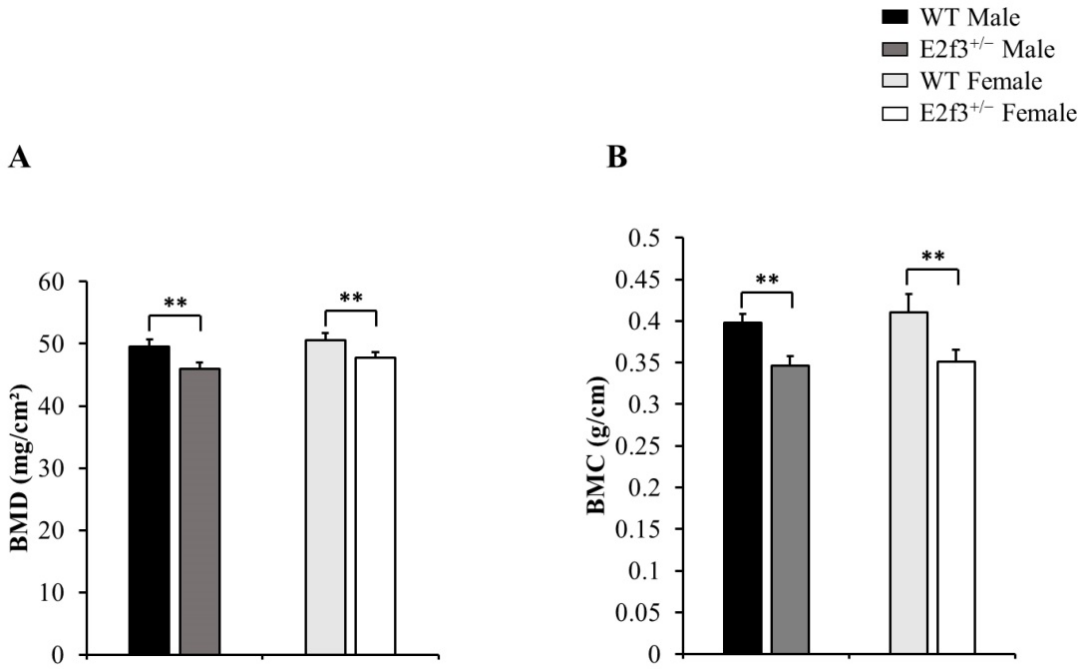
*E2f3*^+/-^ male and female mice showed reduced BMD and BMC, than WT. (A) BMD was significantly reduced in *E2f3*^+/-^ Male (dark gray bar, n = 7) and Female (white bar, n = 8) mice, compared to their control groups (black bar for WT Male; n = 8, light gray bar for WT Female; n = 8). (B) BMC was similarly reduced, in both genders of *E2f3*^+/-^ mice. **P<0.01.

**Figure 3:**
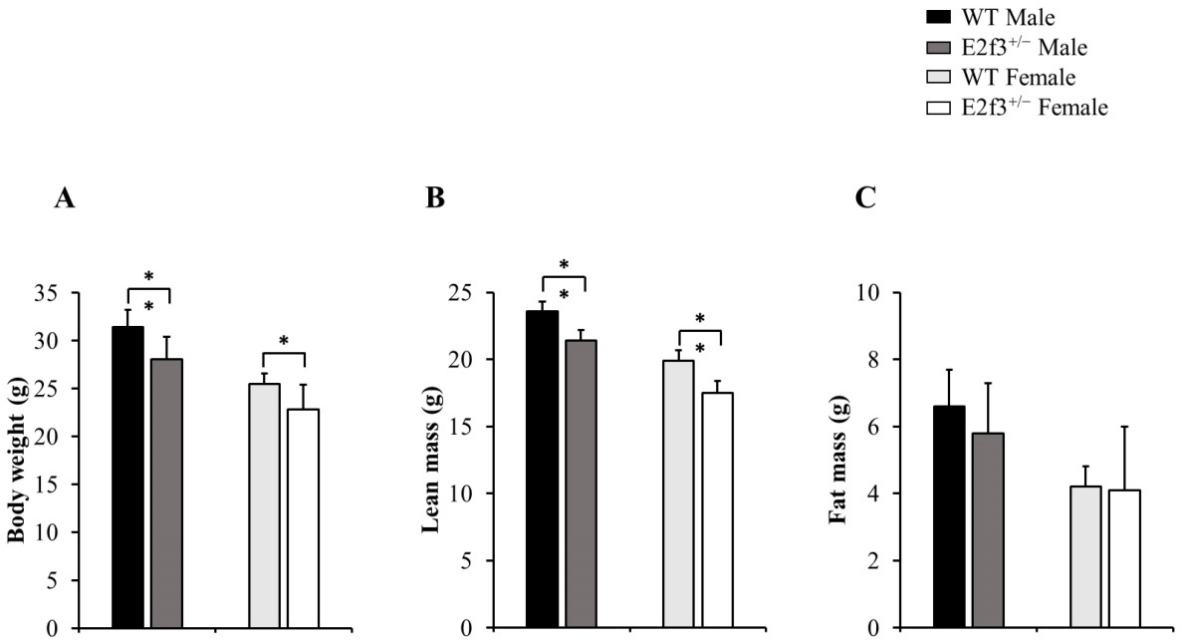
Body composition of 14-week-old *E2f3*^+/-^ mice (both genders) has lower body weight, lean mass than WT strain. Body weight (A) and lean mass (B) were significantly lower in *E2f3*^+/-^ Male (dark gray bar, n = 7) and Female (white bar, n = 8) mice, than their control groups (black bar for WT Male; n = 8, light gray bar for WT Female; n = 8). (C) However, fat mass between WT and *E2f3*^+/-^ was not significantly different in both genders. *P<0.05 and **P<0.01.

Also, body composition of these mice measured by DEXA indicated that *E2f3*^+/-^ mutant mice had lower bone mineral density (BMD) and content (BMC) and lower lean mass, indicating poor bone and muscle development in* E2f3*^+/-^ mutant mice. Three skeletal parameters (BMD, BMC and bone area) of all *E2f3*^+/-^ mutant mice were significantly lower (P<0.01) than wild littermate animals, but there were no morphological abnormalities in the bone when observed in X-ray records (Fig 2 A-B and data not shown). Consistent with reduced body weight and skeletal parameters, *E2f3*^+/-^ mice showed significantly (P<0.01) poor lean mass (male: 21.4±0.8g, female: 17.5±0.9g) than the control groups (male: 23.6±0.7g, female: 19.9±0.8g). However, fat mass did not correlate with *E3f3* mutation (Fig. 3).

### Analysis of muscle strength in male/female E2f3^+/-^ and wild mice

**Figure 4:**
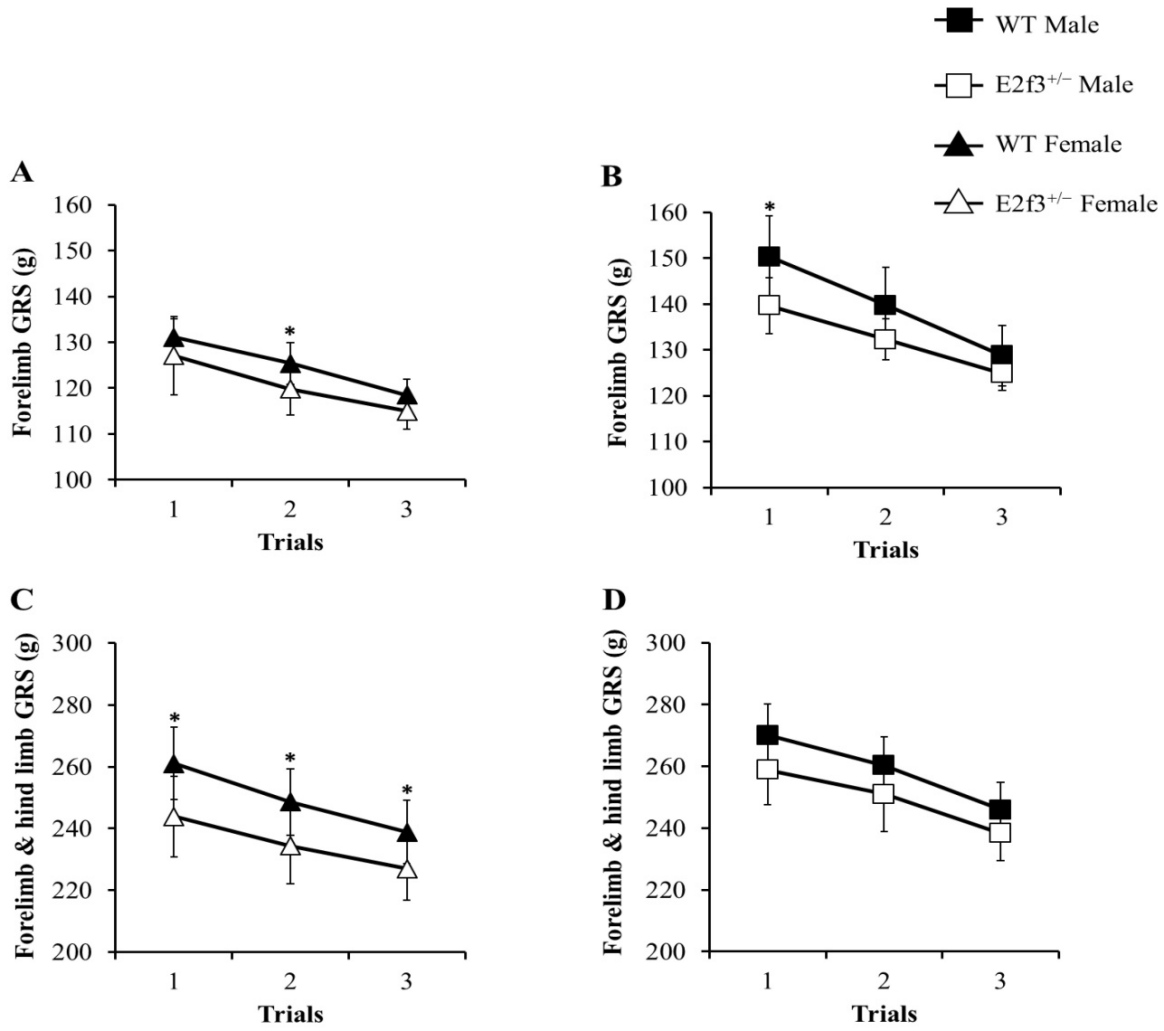
Grip strength (GRS) analysis of WT and *E2f3*^+/-^ mice. Forelimb GRS was observed lower in female (A) and male (B) *E2f3*^+/-^ mice (filled square for WT Male; n = 8, open square for *E2f3*^+/-^ Male; n = 7, filled triangle for WT Female; n = 8 open triangle for *E2f3*^+/-^ Male; n = 8). (C) Forelimb and hind limb combined GRS of female *E2f3*^+/-^ mice was significantly low, compared to their control group in all trials. (D) Forelimb and hind limb combined GRS of male *E2f3*^+/-^ mice was weaker, than their control group like in female, but was not statistically significant. *P<0.05.

The grip strength (GRS) test was performed, to measure neuromuscular function as maximal muscle strength. In all trials, *E2f3*^+/-^ mice showed weak grip strength, compared to WT mice (Fig. 4). Forelimb grip strength of the male *E2f3*^+/-^ mice was significantly lower, than that of the wild mice at trial 1 and on average. Similarly, female *E2f3*^+/-^ mice showed weaker strength, than the wild group at trial 2. In the case of combined forelimb/hind limb strength, female *E2f3*^+/-^ mice exhibited significantly lower strength than the wild type group in all three trials. However, in the male, the values were also lower but not statistically significant.

### E2f3^+/-^ mice have abnormal heart weight than wild mice

**Figure 5:**
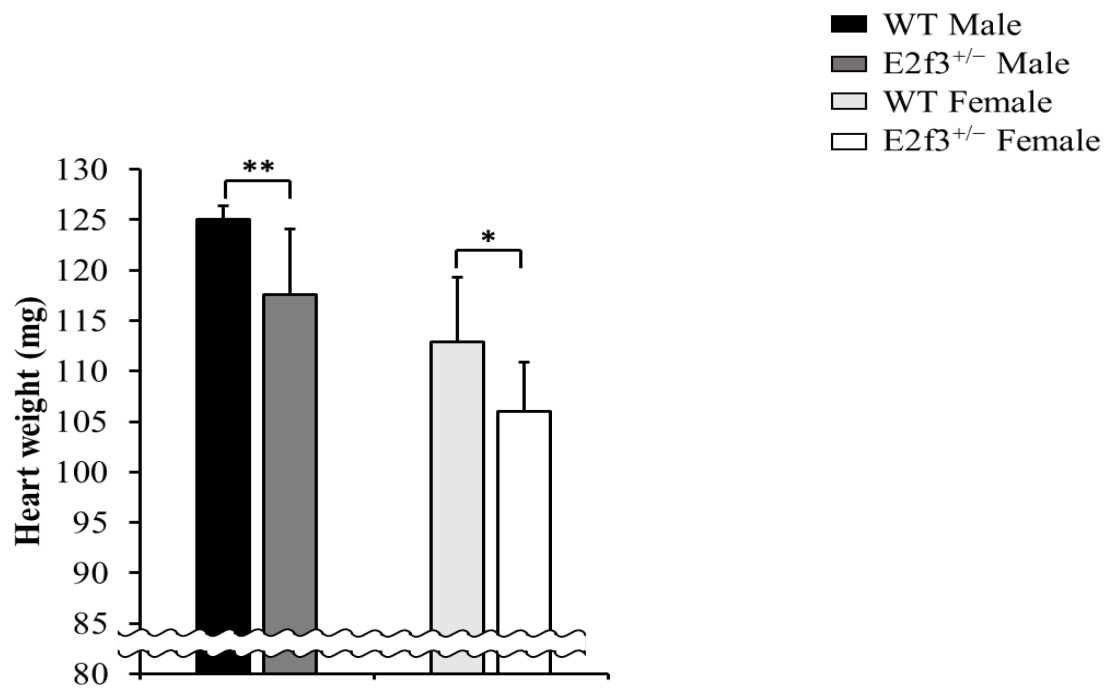
Heart weight analysis of *E2f3*^+/-^ mice, at necropsy. Heart weight was significantly reduced in *E2f3*^+/-^ mice (dark gray bar for *E2f3*^+/-^ Male; n = 7, white bar for *E2f3*^+/-^ Female; n = 8), compared to WT animals (black bar for WT Male; n = 8, light gray bar for WT Female; n = 8). *P<0.05 and **P<0.01.

Full necropsy at age 16 weeks was performed. There was no obvious abnormal gross pathology except in heart weight. The *E2f3*^+/-^ mice exhibited significantly lower heart weight (male: 117.59±6.42mg, female: 106.05±4.88mg) than wild mice (male: 125.01±1.36mg, female: 112.86±6.51mg), and it is parallel to light body weight and lean mass (Fig. 5).

### FACS analysis and assessment of hematological parameters

We also performed hematological test and FACS analysis to assess hematological and immunological problems, but there were no abnormal findings in these analyses when compared with those in the wild type mice (Table 2 and data not shown).

## Discussion

In this study, we explored the function of the E2F3 gene in normal development of mice. E2F3 that regulates cell cycle progression is required for development of most tissues and organs in the body. Because of their importance in development and other important processes, here we conducted several *in vivo* analyses to identify abnormalities in *E2f3*^+/-^ mice, which pave the way for further research.

Herein, we showed that E2F3 is the pre-requisite factor for normal development in mice. In a previous study that investigated viability of the E2F3 mutant mouse, all *E2f3^-/-^* 129/Sv embryos were dead. However, *E2f3^-/-^* mice with C57BL/6 × 129/Sv mixed background survived partially, suggesting that *E2f3* is an essential gene for embryonic viability in a strain-specific manner 19. Our data also indicate that presence of E2F3 in C57BL/6N background is essential for embryo survival and that the amount of E2F3 is also important for the normal development of bones and muscles in mice. It might be possible that incidence of viability of E2F3 embryo depends on the genetic background of mice.

A previous study demonstrated that E2F3, plays a pivotal role in normal cardiac development. A small fraction of *E2f3^-/-^* neonates showed normal heart. However, these animals ultimately died from defects in the cardiac muscle, and as a result of congestive cardiac failure 32. Our observation that *E2f3*^+/-^ mice had lower heart weight compared with that of littermate wild mice, may be associated with the partial deficit of E2F3 that may affect poor heart muscle development in *E2f3*^+/-^ mice.

On the contrary heterozygous mutant, *E2f3^+/-^*mice had shown remarkable significant phenotypes. *E2f3* is not an allelic exclusive gene, and is expressed more in *E2f3^+/+^* cells than *E2f3^+/-^* cells 33. Growth retardation with skeletal imperfection of *E2f3*^+/-^ mice, suggest that *E2f3* expression level is crucial for postnatal musculoskeletal development. E2F3b has been characterized as an essential player, in myogenic differentiation and development 13. Reduced lean mass, grip strength, and heart weight of *E2f3^+/-^* mice, may be associated with specific function of E2f3b in myogenesis. Unlike muscle development, the specific role of E2f3 in osteogenesis is unknown. Small bone area and low BMD and BMC of *E2f3^+/-^* mice strongly suggest E2f3 has a unique function in bone development. Earlier studies have shown that activator E2F transcription factors share a binding motif, and that loss of E2F3 activity triggers compensation effects of other activator of E2Fs 34-37. Our results imply that compensation effects are insufficient to make up for a unique function of E2F3 in muscle and bone development, and leads to growth retardation.

Previous studies have demonstrated the importance of the E2F family in hematopoiesis 5, 38, 39. Individual knock-out of *E2f1*, *E2f2*, and *E2f4* causes' hematopoietic impairment, and also, *E2f1-3* triple knock-out leads to tremendous decline of bone marrow cellularity and CD11b^+^ myeloid cell count 5, 38-41. However, individual Mx-Cre; *E2f3*^fl/fl^ mice exhibited normal bone marrow cellularity 5. In this study, *E2f3^+/-^*mice showed normal blood cell count/hematocrit level including CD11b^+^ cells, and is in contrast to musculoskeletal imperfection. It seems that E2f3 has functional redundancy with other E2Fs in hematopoietic development, and consequently *E2f3* deletion does not cause hematopoietic defects.

Also, because E2F3 contributes to neural development, more explanation is needed as to if E2F3 mutation affects neural functions or not 18, 42.

In this study, we found that complete ablation of *E2f3* was fully penetrant in the pure C57BL/6N background, and that *E2f3^+/^*^-^ mouse embryo developed normally without fatal disorders. However, they exhibited reduced body weight, growth retardation, skeletal imperfection, and poor muscle condition, but no detectable hematopoietic defects. Findings suggest that E2F3 has a pivotal role in muscle and bone development. However, it remains to be elucidated how E2F3 works, in different tissues or environments.

## Figures and Tables

**Table 1:** Analysis of progeny, arising from mating of *E2F3^+/-^* mice in the C57BL/6N background.

	*E2f3*^+/+^	*E2f3*^+/-^	*E2f3*^-/-^
Number of pups	41	77	0
Expected ratio	1	2	1
Observed ratio	1	1.88	0

**Table 2:** FACS data analysis, for major cell types.

Parameters (%)	Male		Female	
	WT	*E2f3*^+/-^	WT	*E2f3*^+/-^
T cells	15.83±0.51	17.07±2.87	25.23±1.98	20.83±1.00*
CD4 T cells	10.80±0.26	12.03±1.88	14.37±1.1	12.27±0.75*
CD8 T cells	4.35±0.24	4.94±0.98	8.39±0.76	6.51±0.2*
B cells	40.67±2.85	40.87±2.75	38.4±2.6	38.27±1.29
NK cells	2.31±0.05	2.39±0.07	1.88±0.24	2.17±0.26
NKT cells	0.59±0.05	0.76±0.11	0.98±0.11	0.95±0.03
Neutrophils	2.14±0.32	2.28±0.34	1.77±0.34	1.66±0.29
Monocytes	0.57±0.11	0.68±0.12	0.49±0.09	0.39±0.05
Macrophages	1.68±0.31	1.66±0.31	1.31±0.42	1.5±0.21
